# Effect of Aging on the Quality of Breast Meat from Broilers Affected by Wooden Breast Myopathy

**DOI:** 10.3390/ani11071960

**Published:** 2021-06-30

**Authors:** Rodrigo Fortunato de Oliveira, Juliana Lolli Malagoli de Mello, Fábio Borba Ferrari, Rodrigo Alves de Souza, Mateus Roberto Pereira, Erika Nayara Freire Cavalcanti, Erick Alonso Villegas-Cayllahua, Heloisa de Almeida Fidelis, Aline Giampietro-Ganeco, Maísa Santos Fávero, Pedro Alves de Souza, Hirasilva Borba

**Affiliations:** 1 Department of Technology, Universidade Estadual Paulista—UNESP, N/n, Professor Donato Castellane Access Road, Rural Zone, Jaboticabal 14884-900, SP, Brazil; julianalolli@zootecnista.com.br (J.L.M.d.M.); fbf_zoo@hotmail.com (F.B.F.); mateusscj2012@hotmail.com (M.R.P.); erikanayarac@gmail.com (E.N.F.C.); evillegasc22@gmail.com (E.A.V.-C.); heloisa.a.fidelis@gmail.com (H.d.A.F.); maisa.sfavero@gmail.com (M.S.F.); p.souza@unesp.br (P.A.d.S.); 2 Faculty of Animal Science and Food Engineering, University of São Paulo—USP, 225, Duque de Caxias Norte Avenue, Pirassununga 13635-900, SP, Brazil; rodrigo.zootecnista@gmail.com (R.A.d.S.); giampietroganeco@gmail.com (A.G.-G.)

**Keywords:** chemical composition, chicken breast meat, meat quality, muscle abnormalities, myodegeneration, *pectoralis major* muscle, softness, tenderness

## Abstract

**Simple Summary:**

The study of meat quality of modern birds and their respective myopathies is important to understand the responses of myopathy to the meat quality of these birds. Aging enters as an alternative to avoid losses that this myopathy generates in the poultry industry, with discards of chickens affected by the different degrees of myopathy in wooden breast. The constant genetic evolution that birds have suffered and still suffer during these years is the most plausible cause of the onset of this myopathy.

**Abstract:**

This study aimed to evaluate the effects of aging on the quality of breast meat from broilers affected of wooden breast. Samples that were classified as normal (control), moderate (hardness verified only in one region of breast fillet), and severe (hardness verified in all the extension of breast fillet) were evaluated fresh and after three and seven days of aging. Normal samples and samples with a moderate degree of myopathy showed greater water-holding capacity, which may benefit the processing industry of poultry meat. During the aging process, increase was observed in total collagen concentration (from 0.41% in normal samples to 0.56% in samples with severe degree). Samples of chicken breast affected by moderate degree showed higher myofibril fragmentation index (MFI = 115) than normal chicken samples (95.65). Although chicken samples affected with severe degree of wooden breast myopathy are more tender than normal chicken breasts, they produce more exudate, which can be detrimental to the processing of poultry meat. The aging process may improve the reduction of cooking weight loss and protein loss in exudation of broilers’ breasts affected by wooden breast myopathy.

## 1. Introduction

Over the past 25 years, the market for chicken meat has largely increased when compared to other animal products. This may be attributed to health, absence of cultural and religious limitations to its consumption, production efficiency, and growth of human population [1].

Genetic advances in chicken meat growth and yield have resulted in muscle changes in these birds, with these being the subject of current research. However, muscle lesions called myopathies have been observed in fast-developing chicken lines, and these are only detected at the time of slaughter. These obstacles to the industry, such as deep pectoral myopathy (DPM), one of the earliest myopathies reported in poultry (turkeys, chickens) with changes in color from pink to green on the pectoralis minor [2]; the appearance of white stripes at different degrees of severity [3]; and spaghetti meat that manifests itself as a loss of integrity of the muscle fiber bundles composing the breast muscle itself, which appears mushy and sparsely tight [4], have heightened the need for research on the physical, chemical, and sensory changes genetic progress can introduce into the quality of the produced meat.

One of these changes, wooden breast myopathy (WBM), is an emerging muscle abnormality in chicken breast fillets that gives the meat an unpleasant appearance. The breast meat affected by this condition is characterized by palpable hardness, full or partial muscle stiffness, and significant changes in the quality characteristics in comparison to normal fillets [5]. In addition, the fillets affected by WBM often exhibit more white stripes on their surface, gelatinous-viscous surface fluid, and petechial hemorrhagic lesions [6]. The incidence of this myopathy has been reported in different countries such as Brazil, Italy, Finland, The United States, and The United Kingdom [7,8]. Reports from the northeast of Brazil show that the proportion of chicken breasts affected by this myopathy is between 10% and 20%. In Italy, this proportion reaches 50% of the breasts, whereas in the United States, the incidence is 85% of the collected samples (42% of severe degree), considering that the classification criteria can vary widely between surveys [9].

Wooden breast myopathy impairs the quality and appearance of breast fillets. The fillets affected by the severe degree of the myopathy can be rejected at the inspection stage still at the slaughterhouse or later during processing. As the chicken breast is the cut with the highest added value, its rejection causes significant economic losses, which increase the industry interest in improving its utilization. For this reason, the chemical changes caused by the myopathy such as increased moisture and lipid content, reduced protein concentration, and water-holding capacity [10,11,12] must be approached. Other concerns include the potential reduction of animal welfare of the affected poultry and ethical issues related to the sentencing of considerable amounts of chicken meat [13,14].

The aging process is a method used to increase the meat tenderness that consists of keeping the meat refrigerated at temperatures close to 0 °C for a period sufficient to make it not only tender, but also to improve other sensory characteristics, especially texture and aroma, significantly influencing its palatability [15]. In this process, significant changes take place in the muscle structure due to the resolution of rigor mortis, and the tenderness is the result of the efficiency with which enzymatic degradation occurs to deconstruct the compacted myofibrils during the rigor mortis process. Thus, as wooden breast is a muscular change that results in a hardened breast meat, a viable alternative for the commercialization of this type of meat might be the use of smoothing techniques such as aging, which can improve its quality, although it is not a common practice in the poultry industry. Some studies were conducted to analyze the effects of storage over the texture [16] and meat proteolysis [17].

Considering that WBM affects the poultry industry globally, research should be conducted to elucidate the dynamics of this disorder as well as determine its effects on poultry meat quality and alternative to avoid losses that this myopathy generates in the poultry industry. This study aimed to evaluate the possible effects of aging for up to seven days on the quality of breast meat of broilers affected by different severity degrees of wooden breast myopathy.

## 2. Materials and Methods

### 2.1. Sample Collection and Experimental Procedure

The experiment was performed in the city of Jaboticabal (State of São Paulo, Brazil), located at 21°17′05″ S latitude and 48°17′09″ W longitude, with an elevation of 590 m.

We used 180 samples (pairs of breasts from 180 chickens) of breast meat from male Cobb MX broilers raised in the traditional intensive system and slaughtered at 45 days old. Samples were obtained from a commercial slaughterhouse (São Paulo, SP, Brazil), which is inspected the by the Federal Inspection Service. The birds were slaughtered according to the slaughterhouse’s routine using eletronarcosis before bleeding, followed the occurrence of rigor mortis (less than half an hour) and mechanical boning of the breast. Afterwards, the samples were transported to the university’s laboratory under cooling conditions for further quality analyses. The samples with no bones and skin were classified by palpation according to the myopathy severity (moderate (*n* = 60)—hardness verified only in cranial or caudal region of the breast fillet; severe (*n* = 60)—hardness verified in all of the breast fillet extension). Sixty samples of breast fillets classified as normal (absence of myopathy) were also collected and used as control for comparison with the affected samples.

The collected samples were identified, individually weighted, and vacuum packed (Selovac 200-B, Selovac, São Paulo, SP, Brazil) in plastic bags (18 μm) and aged in a BOD incubator (EL101 Eletrolab/250W 3, Eletrolab, São Paulo, SP, Brazil) at 2 °C ± 0.5 °C for three and seven days. The physical analyses described below were performed on non-aged samples (on the day of collection *n* = 60) and after three (*n* = 20 for each severity degree and control group, total *n* = 60) and seven days (*n* = 20 for each degree of severity and control group, total *n* = 60) of aging. For chemical analyses, sub-samples of each studied group were prepared in each sampling period (beginning—no aging and after three and seven days of aging) and frozen (−20 °C) for a period not exceeding 30 days for subsequent analyses.

### 2.2. Physical Analysis

The color (lightness—L *, redness—a *, and yellowness—b *) was determined immediately after boning (in non-aged samples) with the use of a Minolta CR-400 colorimeter (Konica Minolta Sensing, Inc., Osaka, Japan) (configurations: diffuse illumination/angle of view, illuminant D65, specular component included, 8 mm measuring area) calibrated for a white standard. The equipment was positioned at three different sites on the outer surface of the pectoralis major muscle (which was previously in contact with the skin) and also at three different sites on the inner surface of the muscle (which was in contact with the sternum). After three and seven days of aging, samples from the control group and groups affected by moderate and severe degrees of the myopathy were removed from the BOD incubator. After the opening of packages, the samples were exposed to oxygen for 30 min for myoglobin oxygenation [18]. The same procedures described above were adopted in the color evaluation of samples aged for three and seven days.

The meat’s pH was evaluated in triplicate with a digital pH Meter (Testo 205, Testo Inc., Sparta, NJ, USA) provided with a penetration electrode that was inserted into the cranial part of each sample. Water-holding capacity (WHC) was determined on a 2 g sample of the *Pectoralis major* muscle. For this evaluation, the sample was placed between two filter papers and acrylic plates and a 10 kg weight was placed on top of the plate for 5 min. Subsequently, the sample was weighed again to measure the amount of water lost using the following formula: (Final weight × 100)/Initial weight [19].

To evaluate cooking losses (CL), we individually weighed samples of the *Pectoralis major* muscle of similar weights and sizes, then vacuum-packed them and cooked them in a water bath (85 °C) for 30 min. Next, the samples were cooled at room temperature and weighed again to determine cooking loss by the following formula: (Initial weight—Final weight) × 100/Initial weight [20]. Shear force was analyzed in samples of the *Pectoralis major* muscle that had been used for the analysis of cooking loss, using the Warner-Bratzler device [21] and using the MORS device in six different locations on the breast surface (1—cranial portion; 2—median portion; 3—caudal portion). The instruments were coupled to a texture analyzer (TA-XT2i, Stable Micro Systems, Godalming, UK). Samples were cut into strips (1 cm^2^ section area and 3 cm in length) that were positioned with the fibers perpendicular to the Warner–Bratzler device, and sheared. The force required to shear the sample was expressed in newtons.

The weight loss by aging was defined as the difference between initial and final weights of each sample before and after aging, expressed as a percentage. The exudate volume was measured after the package’s opening with a degreed testing tube.

### 2.3. Chemical Analyses

The soluble protein in the exudate was evaluated as described by Hartree [22].

The chemical composition was determined in samples that were unaged and aged for three and seven days after the performing of physical analyses. The samples were lyophilized (SuperModulyo220, Thermo Fisher Scientific Inc., Waltham, MA, USA) and ground for subsequent determination of protein and mineral matter concentrations, according to AOAC [23], Methods 977.14 and 920.153, respectively. The percentage of moisture content was determined by the difference between the weights of the samples before and after lyophilization [23], Method 950.46.

Lipid oxidation was determined as previously described [24] by the analysis of TBARS, expressed in milligrams of malondialdehyde (MDA) per kilograms of sample. Previously ground raw samples weighing 5 g were added to 25 mL of trichloroacetic acid solution. After homogenisation in a Turrax stirring device (MA 102, Marconi Laboratory Equipment Ltd., Piracicaba, SP, Brazil) followed by filtration, an aliquot of 5 mL was pipetted and placed into a test tube containing an additional 5 mL of thiobarbituric acid solution. Tubes were kept in a water bath at 96 °C for 40 min, and, after cooling at room temperature, readings were performed on a spectrophotometer at 538 nm accompanied by a standard curve. The concentrations of total, soluble, and insoluble collagen were quantified by determining the hydroxyproline amino acid according to the methodologies proposed by Carvalho et al. [25]. A total of 5 g of frozen raw meat cut into small pieces in 50 mL falcon tubes was used. A total of 20 mL of distilled water was added to the sample immediately before it was taken to the water bath (80 °C for two hours). Subsequently, the samples were homogenized in Ultra-turrax (Marconi MA102, Marconi Equipamentos Para Laboratórios Ltd.a., Piracicaba, SP, Brazil) (22.000 rpm for 1 min) and centrifuged (Himac CR22N, Hitachi Koki do Brasil Ltd.a., Indaiatuba, SP, Brazil) (4000 rpm for 15 min). The samples were transferred to autoclavable tubes, separating the solid fraction from the liquid fraction. A total of 30 mL of 6N HCl was added to the liquid fraction, and 50 mL of 6N HCl was added to the solid fraction. All samples were hydrolyzed in an autoclave (Phoenix Luferco AV-75 Plus, Phoenix Industria e Comercio de Equipamentos Científicos Ltd.a., Araraquara, SP, Brazil) for 4 h (120 °C and 1 atm). After hydrolysis, the pH of all samples was adjusted to 6.0 (with pH Tecnometer MS Tecnopon mPA-210, MS Tecnopon Equipamentos Especiais Ltd.a., Piracicaba, SP, Brazil) using NaOH 2N. The solid fraction was filtered into a 250 mL volumetric flask, the liquid fraction was filtered into a 100 mL volumetric flask, and the flask’s volume was filled with distilled water. Afterwards, a 10 mL aliquot was removed from each filtered sample and transferred to a new volumetric flask (100 mL for solid fraction and 50 mL for liquid fraction). The volume of the flasks was filled with distilled water once again. Then, a 2 mL aliquot of the solid and liquid fractions was transferred in duplicate to test tubes, to which 1 mL of oxidation reagent (Chloramine-T 1.41%) and 1 mL of color reagent (10 g of p-dimethylaminobenzaldehydo in 35 mL of 60% perchloric acid and 65 mL of isopropanol) were added. Finally, the samples were taken to water bath (15 min, 60 °C) and read in a spectrophotometer (Shimadzu UV-1800, Shimadzu Corporation, Kyoto, Japan), with λ equal to 560 nm. Results for the concentration of soluble collagen were obtained from the liquid fraction of the sample, and results for the concentration of insoluble collagen were obtained from the solid fraction. The standard curve was analyzed using a solution with known concentration of hydroxyproline. The calculations of collagen concentrations were performed according to the formulas, where F is equal to 8.33 (mean of the absorbance values equivalent to 1 mg of hydroxyproline obtained from the standard curve) and 7.14 is the conversion factor of hydroxyproline to collagen.

The MFI was determined as described by Culler et al. [26]. From each frozen sample, we obtained 3 g subsamples that were chopped with a scalpel to remove any visible fat and connective tissue. Subsequently, samples were homogenized in a Turrax stirring device (MA 102, Marconi Laboratory Equipment Ltd., Piracicaba, SP, Brazil) for 1.5 min, with 30 mL of extraction buffer. Then, the homogenate was centrifuged at 14,400× *g* for 15 min and 4 °C. After the supernatant was discarded, the precipitate was dispersed in 30 mL of extraction buffer, stirred with a glass rod, and centrifuged again; this operation was repeated once more. After the supernatant was discarded, 15 mL of extraction buffer was added to the precipitate, and the resulting suspension was filtered using a polyethylene strainer to remove connective tissue. The protein concentration of the myofibril suspension was determined by the Biuret method as described by Gornall et al. [27]. An aliquot from the suspension of myofibrils was diluted with extraction buffer to a protein concentration of 0.5 ± 0.05 mg/mL. The diluted suspension of myofibrils was stirred and, shortly thereafter, its optical density was measured in a spectrophotometer at 540 nm. The MFI was calculated according to the following formula: MFI = optical density × 200.The sarcomere length was determined as described by Cross et al. [28]. From each thawed sample, we obtained 0.5 g of sub-samples that were withdrawn with a scalpel and placed into 50 mL falcon tubes. Afterwards, 15 mL of potassium iodide (13.28 g/1 L of distilled water) and 15 mL of potassium chloride (5.96 g/1 L of distilled water) were added. Subsequently, the samples were homogenized in Ultra-Turrax at 15,000 rpm for 30 s. The slides were prepared by dripping one drop of the homogenate into a microscope slide, which was covered by a cover slip. The readings were performed under a phase contrast microscope at 1000× magnification (objective of 100×, ocular of 10×) after a drop of immersion oil was applied on the coverslip. The sarcomere length was determined by the microscope (Novel BM2100) expressed in micrometers.

### 2.4. Statistical Analysis

The samples of breast meat were distributed in the factorial completely randomized experimental design (3 × 3) with 9 treatment groups and 20 repetitions; the samples of breast meat were considered one experimental unit, 20 samples of breast meat per treatment, totaling 180 units.

The resulting data physico-chemical analyses were submitted to the Lilliefors test, verifying the normality of distribution. Afterward, the data were submitted to analysis of variance (F test), at 5% probability, using the general linear models procedure of the Statistical Analysis System (SAS, Institute Inc. 2002–2003, Cary, NC, USA). The Tukey test assessed the significance of the differences between the results at *p* < 0.05.

## 3. Results

### 3.1. Meat Color and pH

Chicken meat affected by wooden breast myopathy, regardless of the severity degree, showed higher (*p* < 0.0001) pH than normal chicken meat (Table 1).

The aging process showed an effect (*p* = 0.002) on the breast meat’s pH, whose value decreased from 6.14 ± 0.02 (beginning of aging) and 6.12 ± 0.02 (after three days of aging) to 6.04 ± 0.02 (after seven days of aging), regardless of the myopathy (Table 1).

The meat affected by the severe degree of the myopathy showed greater (*p* < 0.0001) L * on the outer surface than samples classified as normal or moderate degree. Regardless of myopathy involvement, the value of L * on the outer surface decreased (*p* = 0.021) after three days of aging.

There was a significant interaction between the severity degree and aging period for the variables a * (analyzed on inner and outer surfaces of samples) and b * (analyzed on the outer surface of samples) (Table 1). There was no effect (*p* > 0.05) of myopathy or aging process on outer b * and inner L * values.

The a * values are similar among muscle samples at the start (interaction effect), but samples affected by the myopathy that were aged for three and seven days presented lower a * value (*p* = 0.0003) on the fillets’ inner surface, compared to normal samples. During aging, both normal and myopathic samples showed an increase (*p* < 0.0001) in the a * value on fillet’s inner surface. Regarding the yellowness, samples that were unaged and affected by the severe degree of the myopathy showed higher b * value than normal samples or samples affected by the moderate degree. After three and seven days of aging, there was no variation of b * value among the samples affected by different severity degrees of the myopathy. Considering each severity degree during the aging process, normal samples presented increased yellowness; the b * value of samples with moderate degree was not influenced by aging. Samples affected by the severe degree of the myopathy showed decrease in yellowness. Regarding the redness of the samples’ outer surface (the one that was previously in contact with the breast skin) along the aging process, an increase (*p* < 0.0001) of a * value was verified in all fillets’ treatments.

### 3.2. Water Retention Capacity, Weight Loss by Cooking, and Shear Force

There was no significant interaction between the severity degree of the myopathy and aging period for water-holding capacity and shear force by Warner–Bratzler and MORS devices on cooked breast samples (Table 2). However, an interaction (*p* < 0.05) between cooking weight loss samples was observed.

Samples from broilers with severe degree of WBM had higher (*p* = 0.0002) WHC and breasts affected with myopathy (regardless of degree) had lower (*p* < 0.0001) CL than meat from normal broilers. However, samples with WBM showed lower (*p* < 0.05) shear force using both the Warner–Bratzler device (severe degree only) and the MORS device (independent of the grade) for analysis, when compared to normal samples. An increase (*p* = 0.0002) of WHC was observed after three days of aging and a reduction (*p* < 0.0001) of CL in samples of moderate degrees (at seven days of aging) of myopathy. During the aging process, there was a reduction (*p* < 0.05) in the shear force and, consequently, an increase in softness.

### 3.3. Storage Weight Loss, Exudate Volume, and Soluble Protein in the Exudate

Samples affected by wooden breast myopathy presented higher (*p* < 0.05) weight loss and exudation (Table 3) due to their lower water-holding capacity (Table 2).

Sample weight loss increased from 3.15% (normal samples) to 3.89 and 3.72% (samples affected by the moderate and severe degree of the myopathy, respectively) alongside the increase in exudate volume from 0.016 (normal samples) to 0.025 and 0.022 mL/g (samples affected by the moderate and severe degree of the myopathy, respectively). Regarding the soluble protein in the exudate, there was significant interaction between the severity degree of wooden breast myopathy and aging period (Table 3). The decrease in soluble protein concentration was verified at seven days of aging in samples affected by the severe degree of WMB, which was also confirmed by the decrease in crude protein after the same period (Table 5). An increase (*p* < 0.05) of WLM and exudate volume was observed after seven days of aging.

### 3.4. Collagen, Myofibrillar Fragmentation Index, and Sarcomere Length

The aging increased (*p* < 0.001) the myofibrillar fragmentation index (MFI) of the samples (Table 4), and the samples with moderate degree of WBM showed a higher MFI, followed by normal samples and samples with severe degree of WBM.

During the aging process, the soluble collagen content increased (Table 4). The aging did not affect the insoluble collagen content but affected the total collagen content in chicken samples affected by the severe degree of WBM.

The increase in sarcomere length was observed during the aging process in normal chicken samples, and its reduction was observed in samples affected by the severe degree of wooden breast myopathy (Table 4). The severe degree samples, with or without aging, showed greater sarcomere length when compared to normal chicken samples and moderated degree samples.

### 3.5. Chemical Composition and Lipid Oxidation

There was an interaction (*p* < 0.05) between severe degree of WBM and aging period for the amount of protein, moisture content, and lipid oxidation (Table 5).

Chicken samples with moderate and severe degree of WBM showed lower protein concentrations and higher moisture content than normal chicken samples at the start. There was decrease in the amount of protein in samples affected by wooden breast and samples with moderate degree of myopathy and maintenance of moisture content in normal samples and samples with moderate and severe degree of WBM due to the aging process.

There was no interaction (*p* > 0.05) between the severe degree of WBM and aging period for the amount of ash. There was a decrease (*p* < 0.0001) of ash concentration from 1.59% (normal samples) to 1.48% and 1.17% in samples affected by moderate and severe degrees of the myopathy, respectively. In addition, there was a reduction (*p* = 0.002) of ash concentration throughout the aging process from 1.57% (beginning) to 1.40% and 1.27% after three and seven days of aging, respectively.

Samples affected by the severe degree of WBM showed higher variation in lipid oxidation values after seven days of aging compared to normal chicken samples.

## 4. Discussion

Wooden breast myopathy had a significant effect on the pH values of chicken breast meat, which was higher than those found in normal chicken meat [29,30]. In detail, the higher final pH value found in fillets with wooden breast myopathy is probably due to the decrease of glycolytic potential and changes in the metabolic pathways [31,32].

The higher values of lightness and yellowness found in samples affected by wooden breast myopathy may be explained, respectively, by changes in the muscular tissue after “tissue degeneration”, which causes greater moisture in the muscles affected by the low water retention capacity, and severe fibrosis found in the fillets [30,33,34]. Lightness may be affected by the water-holding capacity [35] because, during aging, cell membranes lose integrity due to proteolysis; as exudate production increases, the amount of water on the sample surface also increases, and so does the reflection of the incident light [36], which increases lightness.

It is likely that the storage temperature (2 °C ± 0.5 °C) was not enough to prevent the oxidation of myoglobin of the meat, which may have influenced the decrease in a * value on the inner surface of chicken breast affected by the myopathy when compared to normal chicken meat. During aging, an increase in the concentration of free radicals may promote the oxidation of myoglobin molecules, reducing the redness [37]. However, red coloring of breast meat increased with aging, regardless of the myopathy degree.

According to previous studies [11,33], samples affected by wooden breast myopathy exhibited a drastic decrease in water-holding capacity. A similar response was observed when measuring cooking weight loss, with the differences disappearing at the end of the seven-day aging period. According to the same authors, the main factor that contributes to the decrease in meat quality is the decrease in water-holding capacity, which could be associated with the characteristic hardness of the cuts with this myopathy, but that was not observed in this study. The increase in water-holding capacity may result from increased pH and enzymatic degradation of myofibrillary structure [38,39]. This effect was not pH-dependent, as the samples affected by the severe degree in the myopathy showed higher pH than normal chicken samples. In addition, after 3 days of aging, the increase in water-holding capacity may have influenced the decrease in the lightness of chicken samples within 3 days of aging. The losses by cooking, resulting in less succulent and less tender meats [40], were lower in normal chicken samples. The lower water-holding capacity in breasts with WBM severe caused a greater release of exudate in the meat, which consequently reduced the amount of protein in the exudate and in the meat after seven days of aging.

Our MFI results of the moderate degree of WBM contrast with previous findings by Oliveira et al. [41]. Myofibrillar fragmentation index increases continuously with aging of ruminant meat [42], in which there is a direct relationship between MFI and tenderness, with higher values of MFI correlating with lower shear force and greater tenderness [26,43]. In this context, there are no established patterns for MFI in chicken meat, which makes it more difficult to relate it to tenderness [44].

Regarding total collagen, the results indicated that aged or unaged myopathic breast samples showed higher concentrations compared to normal samples, which may increase tissue rigidity and reduce the meat quality. The increase in hardness of the muscle affected by WBM is related not only to the increase in collagen concentration, but also to the structural and extension characteristics of fibrotic collagen, fibril diameter, crosslinking, fibril density, and other structural characteristics [45].

In fact, although greater content of soluble, insoluble, and total collagen was found in wooden breast myopathy (Table 4), cooked samples of broilers affected by the myopathy presented lower shear force for the severe device MORS and only the degree for the device WBTZ (Table 2), and consequently more tenderness when compared to normal chicken breast, possibly due to denaturation and solubilization of collagen crosslinks occurring at temperatures between 53 and 63 °C [46].

The greater sarcomere length in WBM sample severity was explained by the muscle hypertrophy verified in samples affected by the WBM, which induced the lengthening of the sarcomere. The result of sarcomere length (mainly involving aging) suggests that the hardness observed in fillets affected by WBM in other studies occurs due to other circumstances besides the shortening of sarcomeres [47]. Tijare et al. [5] reported that sarcomers affected by WBM tend to be longer than normal breast fillets. The results of shear force (WBTZ and MORS) decreased during aging and also with the severity degree of the sample (Table 2), indicating that the cooked samples affected by WBM are tenderer than normal samples. Some authors believe that there is a direct relationship between sarcomere length and meat tenderness in which the longer the sarcomere length, the greater the meat tenderness [38,48]. This relationship should be used in chicken fillets affected by WBM.

We observed that the involvement of WBM is associated with a significant increase in the percentage of moisture content to the detriment of protein and ash concentrations. The higher moisture content in samples affected by the myopathy may be explained by the presence of edema resulting from inflammatory processes [12], which suggests that the occurrence of wooden breast abnormality led to increased moisture. Aging had no effect on moisture in this research, and similar results are found by Mello et al. [44], who compared the effects of post-mortem aging on breast meat from Cobb 500 and Hubbard ISA broilers. The decrease in the meat’s protein content may be associated to the remarkable changes in the muscle structure and muscle characteristics of pectoralis major affected by WBM [12,49], such as muscle fiber degeneration that contributes to the reduction of water retention capacity, causing greater cooking losses.

High levels of lipid oxidation not only reduce the shelf life of foods, but also affect their sensorial characteristics [44].

## 5. Conclusions

A significant part of the broiler breeding is affected by wooden breast myopathy, regardless of the severity degree, which may imply differences in sensorial quality and impair the product appreciation. In this sense, chicken samples affected by the severe degree of WBM produce more exudate, which can be harmful to the processing of chicken meat. During the aging process, a progressive decrease in cooking weight loss and protein content may be observed. In fact, the protein content in samples affected by the moderate and severe degrees of the myopathy decreased by 3–4%. The aging period for 3 days at 2 °C in breasts with or without myopathy is enough to soften the meat without reducing its quality, which suggests that the aging process may be of great importance for the poultry industry, especially when used for breasts affected by wooden breast myopathy. We suggest further research with alternatives for the use of this type of meat, which is indeed suitable for consumption, but which oftentimes presents a non-standard appearance for commercialization in the form of whole files, and is discarded.

## Figures and Tables

**Table 1:** Mean values for pH, lightness (L *), red (a *), and yellow (b *) intensities of breast meat from broilers affected by wooden breast myopathy and aged for up to 7 days.

	pH	Inner L *	Outer L *	Outer b *	
Severity degree (SD)					
Normal	6.03 ± 0.02 ^A^	59.13 ± 0.62	59.78 ± 0.59 ^A^	2.22 ± 0.45	
Moderate	6.07 ± 0.02 ^A^	59.57 ± 0.55	60.46 ± 0.52 ^A^	2.76 ± 0.37	
Severe	6.20 ± 0.02 ^B^	60.99 ± 0.55	63.55 ± 0.53 ^B^	2.85 ± 0.37	
Aging period (M)					
0 day	6.14 ± 0.02 ^A^	59.91 ± 0.55	62.09 ± 0.52 ^A^	2.76 ± 0.37	
3 days	6.12 ± 0.02 ^A^	59.50 ± 0.58	60.01 ± 0.56 ^B^	2.85 ± 0.37	
7 days	6.04 ± 0.02 ^B^	60.28 ± 0.58	61.68 ± 0.55 ^A^	2.95 ± 0.37	
*p*-value ^†^					
(SD)	<0.0001	0.059	<0.0001	0.638	
(M)	0.002	0.647	0.021	0.059	
(SD × M)	0.11	0.129	0.402	0.57	
Inner a *					
	Aging period (M) (*n* = 20)			*p*-value ^†^	
SD (*n* = 20)	0 day	3 days	7 days		
Normal	0.77 ± 0.23 ^Ab^	2.46 ± 0.26 ^Aa^	2.82 ± 0.26 ^Aa^	(SD)	0.0003
Moderate	0.94 ± 0.22 ^Ab^	1.14 ± 0.22 ^Bb^	1.93 ± 0.22 ^Ba^	(M)	<0.0001
Severe	0.56 ± 0.26 ^Ab^	1.53 ± 0.22 ^Ba^	1.61 ± 0.22 ^Ba^	(SD × M)	0.017
Inner b *					
	Aging period (M) (*n* = 20)			*p*-value ^†^	
SD (*n* = 20)	0 day	3 days	7 days		
Normal	2.76 ± 0.37 ^Bab^	2.22 ± 0.45 ^Ab^	3.60 ± 0.48 ^Aa^	(SD)	0.0002
Moderate	2.95 ± 0.37 ^Ba^	2.85 ± 0.37 ^Aa^	3.81 ± 0.37 ^Aa^	(M)	0.0018
Severe	5.55 ± 0.39 ^Aa^	3.12 ± 0.39 ^Ab^	4.04 ± 0.37 ^Ab^	(SD × M)	0.0078
Outer a *					
	Aging period (M) (*n* = 20)			*p*-value ^†^	
SD (*n* = 20)	0 day	3 days	7 days		
Normal	0.64 ± 0.24 ^Ab^	1.93 ± 0.27 ^Aa^	2.24 ± 0.27 ^Aa^	(SD)	0.638
Moderate	1.18 ± 0.23 ^Ab^	1.13 ± 0.23 ^Ab^	1.95 ± 0.23 ^Aa^	(M)	<0.0001
Severe	0.92 ± 0.23 ^Ab^	1.75 ± 0.23 ^Aa^	1.67 ± 0.23 ^Aa^	(SD × M)	0.037

^†^ Means followed by different letters in columns (uppercase) and in lines (lowercase) were significantly different through the Tukey test (*p* < 0.05). “SD”—severity degree, “M”—aging period.

**Table 2:** Mean values for water-holding capacity (WHC), shear force through Warner–Bratzler (WBTZ) and MORS devices (both in cooked samples), and cooking weight loss (CL) of breast meat from broilers affected by wooden breast myopathy and maturated for up to 7 days.

			WBTZ (N)		MORS (N)
	WHC (%)		(Cooked Sample)		
Severity degree (SD)					
Normal	72.80 ± 0.60 ^A^		23.43 ± 1.31 ^A^		9.15 ± 0.20 ^A^
Moderate	72.22 ± 0.60 ^A^		25.45 ± 1.31 ^A^		8.52 ± 0.20 ^B^
Severe	69.25 ± 0.62 ^B^		18.41 ± 1.31 ^B^		8.46 ± 0.20 ^B^
Aging period (M)					
0 day	70.97 ± 0.61 ^B^		32.01 ± 1.31 ^A^		9.89 ± 0.20 ^A^
3 days	73.47 ± 0.61 ^A^		17.17 ± 1.31 ^B^		8.62 ± 0.20 ^B^
7 days	69.83 ± 0.60 ^B^		18.11 ± 1.31^B^		7.63 ± 0.20 ^C^
*p*-value ^†^					
(SD)	0.0002		0.0010		0.0270
(M)	0.0002		<0.0001		<0.0001
(SD × M)	0.9890		0.2720		0.5260
CL (%)					
	Aging period (M) (*n* = 20)			*p*-value ^†^	
SD (*n* = 20)	0 day	3 days	7 days		
Normal	22.54 ± 0.78 ^Bc^	29.21 ± 0.78 ^Ba^	25.82 ± 0.78 ^Ab^	(SD)	<0.0001
Moderate	25.89 ± 0.82 ^Ab^	30.08 ± 0.78 ^Ba^	19.52 ± 0.78 ^Bc^	(M)	<0.0001
Severe	25.86 ± 0.82 ^Ab^	35.42 ± 0.93 ^Aa^	23.92 ± 0.87 ^Ab^	(SD × M)	<0.0001

^†^ Means followed by different letters in columns (uppercase) and in lines (lowercase) were significantly different through the Tukey test (*p* < 0.05). “SD”—severity degree, “M”—aging period.

**Table 3:** Mean values of weight loss by aging (WLM), exudate, and soluble protein volumes within the exudate of breast meat from broilers affected by wooden breast myopathy maturated for up to 7 days.

		WLM (%)		Exudate Volume (mL/g)	
Severity degree (SD)					
Normal		3.15 ± 0.18 ^B^		0.016 ± 0.001 ^B^	
Moderate		3.89 ± 0.16 ^A^		0.025 ± 0.001 ^A^	
Severe		3.72 ± 0.16 ^A^		0.022 ± 0.001 ^A^	
Aging period (M)					
3 days		2.85 ± 0.14 ^B^		0.015 ± 0.001 ^B^	
7 days		4.33 ± 0.14 ^A^		0.027 ± 0.001 ^A^	
*p*-value ^†^					
(SD)		0.0080		<0.0001	
(M)		<0.0001		<0.0001	
(SD × M)		0.3000		0.2410	
Soluble protein within the exudate (mg/mL)					
	Aging period (M) (*n* = 20)			*p*-value ^†^	
SD (*n* = 20)	0 day	3 days	7 days		
Normal	-	0.115 ± 0.002 ^Aa^	0.120 ± 0.002 ^Aa^	(SD)	0.1412
Moderate	-	0.118 ± 0.002 ^Aa^	0.120 ± 0.002 ^Aa^	(M)	0.6473
Severe	-	0.120 ± 0.002 ^Aa^	0.111 ± 0.002 ^Bb^	(SD × M)	0.0014

^†^ Means followed by different letters in columns (uppercase) and in lines (lowercase) were significantly different through the Tukey test (*p* < 0.05). “SD”—severity degree, “M”—aging period.

**Table 4:** Mean values of myofibrillar fragmentation index (MFI); soluble, insoluble, and total collagen; and sarcomere length of breast meat from broilers affected by wooden breast myopathy maturated for up to 7 days.

	MFI				Soluble Collagen (%)
Severity degree (SD)					
Normal	95.65 ± 2.85 ^B^				0.15 ± 0.01 ^B^
Moderate	115.00 ± 2.96 ^A^				0.20 ± 0.01 ^A^
Severe	72.81 ± 2.85 ^C^				0.19 ± 0.01 ^A^
Aging period (M)					
0 day	79.32 ± 2.85 ^B^				0.16 ± 0.01 ^B^
3 days	99.79 ± 2.90 ^A^				0.17 ± 0.01 ^AB^
7 days	104.35 ± 2.90 ^A^				0.20 ± 0.01 ^A^
*p*-value ^†^					
(SD)	<0.0001				0.0082
(M)	<0.0001				0.0383
(SD × M)	0.1911				0.3790
Insoluble collagen (%)					
	Aging period (M) (*n* = 20)			*p*-value ^†^	
SD (*n* = 20)	0 day	3 days	7 days		
Normal	0.16 ± 0.02 ^Ba^	0.22 ± 0.02 ^Ba^	0.18 ± 0.02 ^Ba^	(SD)	<0.0001
Moderate	0.26 ± 0.02 ^Aa^	0.20 ± 0.02 ^Ba^	0.22 ± 0.02 ^Ba^	(M)	0.2823
Severe	0.24 ± 0.02 ^Aa^	0.31 ± 0.02 ^Aa^	0.31 ± 0.02 ^Aa^	(SD × M)	0.0034
Total collagen (%)					
	Aging period (M) (*n* = 20)			*p*-value ^†^	
SD (*n* = 20)	0 day	3 days	7 days		
Normal	0.29 ± 0.04 ^Bb^	0.46 ± 0.04 ^Aa^	0.33 ± 0.04 ^Cb^	(SD)	0.0014
Moderate	0.43 ± 0.04 ^Aa^	0.37 ± 0.04 ^Aa^	0.45 ± 0.04 ^Ba^	(M)	0.0563
Severe	0.41 ± 0.04 ^Ab^	0.46 ± 0.04 ^Aab^	0.56 ± 0.04 ^Aa^	(SD × M)	0.0052
Sarcomere length (µ)					
	Aging period (M) (*n* = 20)			*p*-value ^†^	
SD (*n* = 20)	0 day	3 days	7 days		
Normal	1.49 ± 0.02 ^Bc^	1.58 ± 0.02 ^ABa^	1.53 ± 0.02 ^Bb^	(SD)	<0.0001
Moderate	1.49 ± 0.02 ^Ba^	1.55 ± 0.02 ^Ba^	1.57 ± 0.02 ^Ba^	(M)	0.0560
Severe	1.68 ± 0.02 ^Aa^	1.63 ± 0.02 ^Ab^	1.64 ± 0.02 ^Aab^	(SD × M)	0.0003

^†^ Means followed by different letters in columns (uppercase) and in lines (lowercase) were significantly different through the Tukey test (*p* < 0.05). “SD”—severity degree, “M”—aging period.

**Table 5:** Mean values of crude protein, moisture content, and lipid oxidation (TBARS) of the breast meat from broilers affected by wooden breast myopathy maturated for up to 7 days.

Crude protein (%)					
	Aging period (M) (*n* = 20)			*p*-value ^†^	
SD (*n* = 20)	0 days	3 days	7 days		
Normal	25.20 ± 0.61 ^Aa^	25.41 ± 0.69 ^Aa^	23.55 ± 0.82 ^Aa^	(SD)	<0.0001
Moderate	23.31 ± 0.58 ^Ba^	24.91 ± 0.58 ^Aa^	18.80 ± 0.65 ^Bb^	(M)	<0.0001
Severe	22.97 ± 0.58 ^Ba^	23.21 ± 0.58 ^Ba^	19.74 ± 0.61 ^Bb^	(SD × M)	0.0401
Moisture content (%)					
	Aging period (M) (*n* = 20)			*p*-value ^†^	
SD (*n* = 20)	0 days	3 days	7 days		
Normal	73.66 ± 0.29 ^Ba^	73.43 ± 0.34 ^Ba^	73.45 ± 0.37 ^Ba^	(SD)	<0.0001
Moderate	74.20 ± 0.29 ^ABa^	73.77 ± 0.29 ^ABa^	73.56 ± 0.30 ^Ba^	(M)	0.0685
Severe	76.41 ± 0.29 ^Aa^	75.49 ± 0.34 ^Aa^	76.64 ± 0.34 ^Aa^	(SD × M)	<0.0001
TBARS (mg MDA/kg of raw samples)					
	Aging period (M) (*n* = 20)			*p*-value ^†^	
SD (*n* = 20)	0 days	3 days	7 days		
Normal	0.61 ± 0.06 ^Ab^	0.60 ± 0.07 ^Ab^	1.28 ± 0.07 ^Ca^	(SD)	<0.0001
Moderate	0.59 ± 0.06 ^Ab^	0.48 ± 0.06 ^Ab^	1.65 ± 0.07 ^Ba^	(M)	<0.0001
Severe	0.65 ± 0.06 ^Ab^	0.56 ± 0.07 ^Ab^	2.21 ± 0.07 ^Aa^	(SD × M)	<0.0001

^†^ Means followed by different letters in columns (uppercase) and in lines (lowercase) were significantly different through the Tukey test (*p* < 0.05). “SD”—severity degree, “M”—aging period.

## Data Availability

This data can be found here: https://repositorio.unesp.br/bit-stream/handle/11449/191219/oliveira_rf_dr_jabo.pdf?sequence=5&isAllowed=y, (accessed on 17 March 2020).

## References

[1] Tallentire C.W., Leinonen I., Kyriazakis I. (2018). Artificial selection for improved energy efficiency is reaching its limits in broiler chickens. Sci. Rep..

[2] Kijowski J., Kupińska E., Stangierski J., Tomaszewska-Gras J., Szablewski T. (2014). Paradigm of deep pectoral myopathy in broiler chickens. Reviews. Worlds Poult. Sci. J..

[3] Mello J.L.M., Souza R.A., Ferrari F.B., Cavalcanti E.N.F., Oliveira R.F., Fidelis H.A., Pereira M.R., Villegas-Cayllahua E.A., Giampietro-Ganeco A., Dutra D.R. (2021). Quality of breast meat from broiler chickens raised in Brazil affected by white striping myopathy. Res. Soc. Dev..

[4] Baldi G., Soglia F., Mazzoni M., Sirri F., Canonico L., Babini E., Laghi L., Cavani C., Petracci M. (2018). Implications of white striping and spaghetti meat abnormalities on meat quality and histological features in broilers. Animal.

[5] Tijare V.V., Yang F.L., Kuttappan V.A., Alvarado C.Z., Coon C.N., Owens C.M. (2016). Meat quality of broiler breast fillets with white striping and woody breast muscle myopathies. Poult. Sci..

[6] Kuttappan V.A., Owens C.M., Coon C., Hargis B.M., Vasquez-Anon M. (2017). Incidence of broiler breast myopathies at 2 different ages and its impact on selected raw meat quality parameters. Poult. Sci..

[7] Ferreira T.Z., Casagrande R.A., Vieira S.L., Driemeier D., Kindlein L. (2014). An investigation of a reported case of white striping in broilers. J. Appl. Poult. Res..

[8] De Brot S., Perez S., Shivaprasad H.L., Baiker K., Polledo L., Clark M., Grau-Roma L. (2016). Wooden breast lesions in broiler chickens in the UK. Vet. Rec..

[9] Petracci M., Soglia F., Madruga M., Carvalho L., Elza I., Estevez M. (2019). Wooden-Breast, White Striping, and Spaghetti Meat: Causes, Consequences and Consumer Perception of Emerging Broiler Meat Abnormalities. Compr. Rev. Food Sci. Food Saf..

[10] Sihvo H.K., Immonen K., Puolanne E. (2014). Myodegeneration with fibrosis and regeneration in the pectoralis major muscle of broilers. Vet. Pathol..

[11] Mazzoni M., Petracci M., Meluzzi A., Cavani C., Clavenzani P., Sirri F. (2015). Relationship between pectoralis major muscle histology and quality traits of chicken meat. Poult. Sci..

[12] Soglia F., Mudalal S., Babini E., Di Nunzio M., Mazzoni M., Sirri F., Cavani C., Petracci M. (2016). Histology, composition, and quality traits of chicken Pectoralis major muscle affected by wooden breast abnormality. Poult. Sci..

[13] Mutryn M.F., Brannick E.M., Fu W., Lee W.R., Abasht B. (2015). Characterization of a novel chicken muscle disorder through differential gene expression and pathway analysis using RNA-sequencing. BMC Genom..

[14] Petracci M., Mudalal S., Soglia F., Cavani C. (2015). Meat quality infast-growing broiler chickens. Worlds Poult. Sci. J..

[15] Lawrie R.A. (1985). Meat Science.

[16] Sun X., Koltes D.A., Coon C.N., Chen K., Owens C.M. (2018). Instrumental compression force and meat attribute changes in woody broiler breast fillets during short-term storage. Poult. Sci..

[17] Soglia F., Zeng Z., Gao J., Puolanne E., Cavani C., Petracci M., Ertbjerg P. (2018). Evolution of proteolytic indicators during storage of broiler wooden breast meat. Poult. Sci..

[18] Ledward D.A., Johnson D.E., Knight M.K., Ledward D.A. (1992). Colour of Raw and Cooked Meat. The Chemistry of Muscle-Based Foods.

[19] Hamm R. (1961). Biochemistry of meat hydratation. Adv. Food Res..

[20] Honikel K.O. (1987). The water binding of meat. Fleischwirttsch.

[21] Lyon C.E., Lyon B.G., Dickens J.A. (1998). Effects of carcass stimulation, deboning time, and marination on color and texture of broiler breast meat. J. Appl. Poult. Res..

[22] Hartree E.F. (1972). Determination of protein: A modification of the Lowry method that gives a linear photometric response. Anal. Biochem..

[23] AOAC International (2011). Official Methods of Analysis.

[24] Vyncke W. (1970). Direct determination of the thiobarbituric acid value in trichloracetic acid extracts of fish as a measure of oxidative rancidity. Fette Seifen Anstrichm..

[25] Carvalho L.T., Owens C.M., Giampietro-Ganeco A., Mello J.L.M., Ferrari F.B., Carvalho F.A.L., Souza R.A., Amoroso L., Souza P.A., Borba H. (2021). Quality of turkeys breast meat affected by white striping myopathy. Poult. Sci..

[26] Culler R.D., Parrish F.C., Smith Junior G.C., Cross H.R. (1978). Relationship of myofibril fragmentation index to certain chemical, physical and sensory characteristics of bovine Longissimus muscle. J. Food Sci..

[27] Gornall A.G., Bardawill C.J., David M.M. (1949). Determination of serum protein by means of the biuret reaction. J. Biol. Chem..

[28] Cross H.R., West R.L., Dutson T.R. (1981). Comparison of methods for measuring sarcomere length in beef semitendinosus muscle. Meat Sci..

[29] Zhuang H., Bowker B. (2018). The wooden breast condition results in surface discoloration of cooked broiler pectoralis major. Poult. Sci..

[30] Baldi G., Soglia F., Laghi L., Tappi S., Rocculi P., Tavaniello S., Prioriello D., Mucci R., Maiorano G., Petracci M. (2019). Comparison of quality traits among breast meat affected by current muscle abnormalities. Food Res. Int..

[31] Zambonelli P., Zappaterra M., Soglia F., Petracci M., Sirri F., Cavani C., Davoli R. (2017). Detection of differentially expressed genes in broiler pectoralis major muscle affected by White Striping—Wooden Breast myopathies. Poult. Sci..

[32] Beauclercq S., Hennequet-Antier C., Praud C., Godet E., Collin A., Tesseraud S., Berri C. (2017). Muscle transcriptome analysis reveals molecular pathways and biomarkers involved in extreme ultimate pH and meat defect occurrence in chicken. Sci. Rep..

[33] Mudalal S., Lorenzi M., Soglia F., Cavani C., Petracci M. (2015). Implications of white striping and wooden breast abnormalities on quality traits of raw and marinated chicken meat. Animal.

[34] Sánchez-Brambila G., Bowker B.C., Zhuang H. (2016). Comparison of sensory texture attibutes of broiler breast fillets with different grades of white striping. Poult. Sci..

[35] Koohmaraie M., Kent M.P., Shackelford S.D., Veiseth E., Wheeler T.L. (2002). Meat tenderness and muscle growth: Is there any relationship?. Meat Sci..

[36] Oliveira F.R., Boari C.A., Pires A.V., Mognato J.C., Carvalho R.M.S., Santos Junior M.A., Mattioli C.C. (2015). Pre slaughter fasting and free-range broilers meat quality. Rev. Bras. Saúde Prod. Anim..

[37] Monahan F.J., Skibsted L.H., Andersen M.L. (2005). Mechanism of oxymyoglobin oxidation in the presence of oxidizing lipids in bovine muscle. J. Agric. Food Chem..

[38] Pearce K.L., Rosenvold K., Andersen H.J., Hopkins D.L. (2011). Water distribution and mobility in meat during the conversion of muscle to meat and ageing and the impacts on fresh meat quality attributes—A review. Meat Sci..

[39] Li P., Wang T., Mao Y., Zhang Y., Niu L., Liang R., Zhu L., Luo X. (2014). Effect of ultimate pH on postmortem myofibrillar protein degradation and meat quality characteristics of Chinese yellow crossbreed cattle. Sci. World J..

[40] Moura J.W.F., Medeiros F.M., Alves M.G.M., Batista A.S.M. (2015). Fatores influenciadores na qualidade da carne suína. Rev. Cient. Prod. Anim..

[41] Oliveira R.F., Mello J.L.M., Ferrari F.B., Cavalcanti E.N.F., Souza R.A., Pereira M.R., Giampietro-Ganeco A., Villegas-Cayllahua E.A., Fidelis H.A., Fávero M.S. (2021). Physical, Chemical and Histological Characterization of *Pectoralis major* Muscle of Broilers Affected by Wooden Breast Myopathy. Animals.

[42] Watanabe A., Daly C.C., Devine C.E. (1996). The effects of the ultimate pH of meat on tenderness changes during ageing. Meat Sci..

[43] Koohmaraie M., Whipple G., Crouse J.D. (1990). Acceleration of postmortem tenderization in lamb and Brahman cross beef carcasses through infusion of calcium chloride. J. Anim. Sci..

[44] Mello J.L.M., Souza R.A., Paschoalin G.C., Ferrari F.B., Machado B.M., Giampietro-Ganeco A., Souza P.A., Borba H. (2017). A comparison of the effects of post-mortem aging on breast meat from Cobb 500 and Hubbard ISA broilers. Anim. Prod. Sci..

[45] Velleman S.G., Clark D.L., Tonniges J.R. (2017). Fibrillar collagen organization associated with broiler wooden breast fibrotic myopathy. Avian Dis..

[46] Soglia F., Gao J., Mazzoni M., Puolanne E., Cavani C., Petracci M., Ertbjerg P. (2017). Superficial and deep changes of histology, texture and particle size distribution in broiler wooden breast muscle during refrigerated storage. Poult. Sci..

[47] Oliveira R.F. (2019). Qualidade da Carne de Peito de Frangos de Corte in Natura e Processada Acometidas Por Peito de Madeira.

[48] Cheng Q., Sun D.W. (2008). Factors affecting the water holding capacity of red meat products: A review of recent research advances. Crit. Rev. Food Sci. Nutr..

[49] Tasoniero G., Cullere M., Cecchinato M., Puolanne E., Dalle Zotte A. (2016). Technological quality, mineral profile and sensory attributes of broiler chicken breasts affected by white striping and wooden breast myopathies. Poult. Sci..

